# [Retracted] Anti‑Semaphorin‑7A single chain antibody demonstrates beneficial effects on pulmonary inflammation during acute lung injury

**DOI:** 10.3892/etm.2023.12165

**Published:** 2023-08-14

**Authors:** Xiao Chen, Hailing Wang, Kui Jia, Hao Wang, Tao Ren

Exp Ther Med 15:2356-2364, 2018; DOI: 10.3892/etm.2018.5724

Following the publication of this paper, it was drawn to the Editor’s attention by a concerned reader that the vWF data presented in Fig. 3F on p. 2361 for the ‘Anti-SEMA-7A’ and ‘Control’ experiments appeared to be duplicates, such that the same data may have been used to show the results from differently performed experiments. Subsequently, the Editorial Office conducted an independent investigation of this paper, and it was found that cellular data featured in both Figs. 3E and 4B in the above paper had already been published in the following respective articles, written by different authors at different research institutes: Sheu J-J, Lee F-Y, Wallace GC, Tsai T-H, Leu S, Chen Y-L, Chai H-T, Lu H-I, Sun C-K and Yip H-K: Administered circulating microparticles derived from lung cancer patients markedly improved angiogenesis, blood flow and ischemic recovery in rat critical limb ischemia. J Transl Med 13: 59, 2015; and Zhang M, Wang L, Dong M, Li Z and Jin F: Endothelial semaphorin 7A promotes inflammation in seawater aspiration-induced acute lung injury. Int J Mol Sci 15: 19650-19661, 2014].

Owing to the fact that the contentious data in the above article had already been published prior to its submission to *Experimental and Therapeutic Medicine,* the Editor has decided that this paper should be retracted from the Journal. The authors were asked for an explanation to account for these concerns, but the Editorial Office did not receive a reply. The Editor apologizes to the readership for any inconvenience caused.

